# Clinical characteristics and longitudinal changes of patients with non-alcoholic fatty liver disease in 2 decades: the NAGALA study

**DOI:** 10.1186/s12876-021-01809-2

**Published:** 2021-05-17

**Authors:** Takuro Okamura, Yoshitaka Hashimoto, Masahide Hamaguchi, Akihiro Obora, Takao Kojima, Michiaki Fukui

**Affiliations:** 1 Department of Endocrinology and Metabolism, Graduate School of Medical Science, Kyoto Prefectural University of Medicine, 465, Kajii-cho, Kawaramachi-Hirokoji, Kamigyo-ku, Kyoto, 602-8566 Japan; 2 Department of Gastroenterology, Asahi University Hospital, Gifu, Japan

**Keywords:** Cohort, Non-alcoholic fatty liver disease, NAFLD, Epidemiology, Trend

## Abstract

**Background:**

In this study, to clarify the evolving background of people with non-alcoholic fatty liver disease (NAFLD), we compared the current prevalence of NAFLD with that of 2 decades ago.

**Methods:**

We included two cohorts. The past cohort was from 1994 to 1997 and included 4279 men and 2502 women. The current cohort was from 2014 to 2017 and included 8918 men and 7361 women. NAFLD was diagnosed by abdominal ultrasonography.

**Results:**

The prevalence of NAFLD increased in both genders throughout these 2 decades (18.5% in the past cohort and 27.1% in the current cohort for men; and 8.0% in the past cohort and 9.4% in the current cohort for women). The prevalence of hyperglycemia increased, whereas the prevalence of low high-density lipoprotein cholesterol levels and hypertriglyceridemia significantly decreased. There was no significant difference in the mean body mass index. Multivariate analysis revealed that the prevalence of obesity and body mass index were significantly associated with the prevalence of NAFLD in both the past and current cohorts.

**Conclusions:**

The incidence of NAFLD significantly increased throughout these 2 decades, and obesity is the most prevalent factor. Thus, body weight management is an essential treatment option for NAFLD.

**Supplementary Information:**

The online version contains supplementary material available at 10.1186/s12876-021-01809-2.

## Introduction

It is well known that non-alcoholic fatty liver disease (NAFLD) is one common cause of chronic liver disease [1], as is the risk of type 2 diabetes and cardiovascular disease concurrently [2, 3]. The prevalence of NAFLD shows a significant variation between countries and regions. Generally, the prevalence in the western countries is 20–40%, whereas in Asian countries 12–30% [4–8], and between 1980 and 2010, mortality associated with chronic liver disease, including NAFLD, increased by 46% worldwide [9].

The environment surrounding NAFLD has continuously been evolving [10, 11]. In Japan, the number of patients with NAFLD has increased to 20–30% because of the shift to a high-fat diet and sedentary lifestyle [12, 13]. Additionally, the age distribution of NAFLD patients indicates that it is more frequent among middle-aged men and older women [14, 15]. It has been reported that the prevalence of NAFLD increased from 12.9% in 1994 to 23.9% in 2004 in Japan [16]. The prevalence of NAFLD is on the rise, with an increasing number of people with obesity and metabolic syndrome.

Therefore, to clarify the evolving background of people with NAFLD, we compared current cohort of patients with cohorts from 2 decades ago. Furthermore, we focused on the metabolic abnormalities of patients with NAFLD.

## Methods

### Study population and design

In Japan, citizens receive a nationwide health checkup program known as Ningen Dokku, which roughly translates to “human dock” (resembling patient checkups to ships being repaired at docks). This nationwide program promotes public health through the detection of chronic diseases, including gastrointestinal illnesses and other types of cancer, and their corresponding risk factors mainly for a primary care population. Most of them are annual medical examinations as instructed by their workplaces, or they receive medical examinations individually. At the time of the examination, the person in charge asked whether or not they agreed to participate in the study, and the study was conducted on subjects who agreed to participate. This routine checkup includes blood and urine examinations, upper gastrointestinal series or gastroesophageal endoscopy, abdominal ultrasonography, and fecal occult blood tests.

We performed a longitudinal cohort study named the NAGALA (NAfld in the Gifu Area, Longitudinal Analysis) to investigate the impact of fatty liver on several aspects of the metabolic syndrome from 1994 onwards [12]. The ethics committee approved the current version of the NAGALA study (IRB number: 2018-09-01). The study design was opt-out sampling. Participants who underwent health checkups at the Asahi University Hospital were contacted to volunteer to take part in this research. Subjects were excluded only if unwilling to participate.

We included two cohorts that were 20 years apart in our study. The past cohort was composed of participants who participated in the health checkup programs from June 1994 to December 1997. The current cohort, which was 2 decades ahead of the past cohort, consisted of participants who participated in the health checkup programs from January 2014 to December 2017.

We excluded patients having liver disease or history of any medication use [12, 17]. Liver disease was indicated if positive for hepatitis B antigen or hepatitis C antibody, or history of known liver disease, including genetic, viral, drug-induced, or autoimmune liver disease [18].

### Data collection and measurements

The methods for data collection and measurements were described in our previous study [17]. Briefly, we used a standardized self-administered questionnaire to collect information not only about the medical history, but also about lifestyle factors, such as alcohol intake, smoking habits, and physical activity [12, 17]. The mean ethanol intake per week was estimated by the amounts and types of alcoholic beverages consumed per week. We divided the patients into three groups: the non-alcoholic group with < 210 g/week for men and < 140 g/week for women (none or minimal alcohol consumption: < 40 g/week, light alcohol consumption: 40–210 g/week for men and 40–140 g/week for women); the intermediate group with 210–420 g/weeek for men and 140–280 g/week for women; and the alcoholic group with ≥ 420 g/week for men and ≥ 280 g/week for women [19].

Regarding the smoking status, we classified the patients into three groups: non-smokers, ex-smokers, and current smokers. The patients were defined as regular exercisers if they participated in any sports activity at least once a week regularly [20]. Body mass index (BMI) was calculated as weight (kg)/height (m) squared. The conventional criteria for Asian obesity (BMI ≥ 25 kg/m^2^) were used [21, 22]. In addition, we defined metabolic abnormality as hypertension (blood pressure > 130/85 mmHg), hyperglycemia (fasting plasma glucose > 5.6 mmol/L), hypertriglyceridemia (serum triglycerides > 1.70 mmol/L), and low high-density lipoprotein (HDL) cholesterol levels (serum HDL cholesterol < 1.03 mmol/L in men and < 1.29 mmol/L in women) [22]. Moreover, NAFLD fibrosis score was calculated with the following formula: − 1.675 + 0.037 × age (years) + 0.094 × BMI (kg/m^2^) + 1.13 × IFG/diabetes (yes = 1, no = 0) + 0.99 × aspartate transaminase (AST)/alanine aminotransferase (ALT) − 0.013 × platelet count (× 10^9^/L) − 0.66 × albumin (g/dL) [23]. FIB4 index was calculated with the following formula: age × AST (IU/L)/platelet count (× 10^9^/L)/√ALT(IU/L) [24].

### Definition of fatty liver and non-alcoholic fatty liver disease

Fatty liver was diagnosed by abdominal ultrasonography performed by a trained technician [25]. Gastroenterologists reviewed the images alone to diagnose fatty liver without referring to other personal data of the patients. In this study, of four known criteria (hepatorenal echo contrast, liver brightness, deep attenuation and vascular blurring), the participants with hepatorenal contrast and liver brightness were diagnosed as having fatty liver. In addition, we defined patients with fatty liver as having NAFLD on the basis of volume of their alcohol intake as < 210 g/week for men and < 140 g/week for women [26].

### Statistical analysis

*P* values ≤ 0.05 were considered statistically significant. We analyzed all data using the JMP software (ver. 13). We divided the participants into men and women. Medians or frequencies of variables were calculated, and continuous variables were presented as the median ± interquartile range (IQR), and categorized variables were presented as a percentage. A chi-square test was performed to assess the statistical significance of differences between the groups for categorical variables. The continuous variables did not follow a normal distribution. Therefore, the Wilcoxon signed-rank test was performed. We performed logistic regression analyses to calculate the unadjusted odds ratios (ORs) and 95% confidence intervals (CIs) of several factors that influence the presence of NAFLD. To examine the effects of several factors on NAFLD, we considered the following factors as independent variables in multivariate logistic regression analyses. Model 1 was adjusted for age, alcohol intake, exercise habits, ALT levels, smoking status, exercise, BMI, systolic blood pressure, fasting plasma glucose, triglycerides, and HDL cholesterol levels. Model 2 was adjusted for age, alcohol intake, exercise habits, ALT levels, smoking status, obesity, hypertension, hyperglycemia, hypertriglyceridemia, and low HDL cholesterol levels.

In addition, the area under the curve (AUC) of several factors, including BMI, systolic blood pressure, fasting plasma glucose, HDL cholesterol, and triglyceride levels, for the prevalence of NAFLD was calculated by the receiver operating characteristic (ROC) curve. Moreover, we sought optimum cut-off value for the prevalence of NAFLD.

## Results

**Fig. 1:**
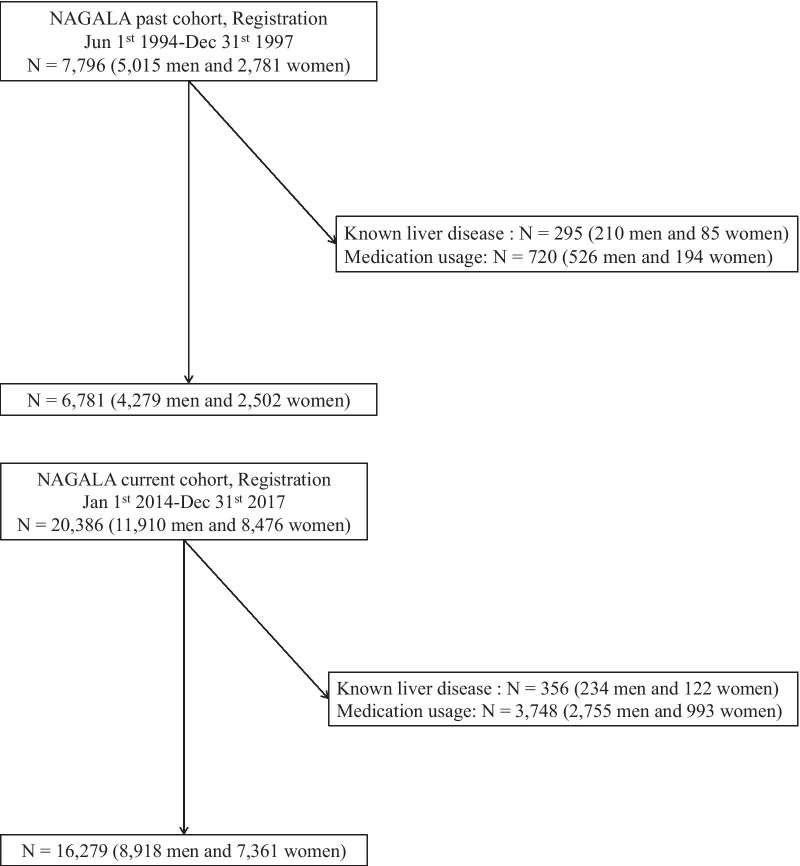
Participant registration. *NAGALA* NAfld in Gifu Area, Longitudinal Analysis, *CKD* chronic kidney disease

The past cohort consisted of 7796 participants (5015 men and 2781 women), whereas the current cohort consisted of 20,386 participants (11,910 men and 8476 women). After excluding patients with known liver disease and history of any medication use, 6781 patients (4279 men and 2502 women) in the past cohort and 16,279 (8918 men and 7361 women) in the current cohort were finally included in our study (Fig. 1).

Table 1 represents the clinical characteristics of our study. The prevalence of NAFLD increased in both men and women throughout these 2 decades (men, 18.5% in the past and 27.1% in the current cohort, *p* < 0.001; and women, 8.0% in the past and 9.4% in the current cohort, *p* = 0.024). Regarding smoking status, the number of current smokers decreased in the 2 decades for both genders. In terms of exercise habits, the number of regular exercisers increased in men, whereas it decreased in women. Regarding alcohol intake, average alcohol intake and number of alcohol drinkers decreased for men. In contrast, the prevalence of alcohol drinkers increased in women, although the average alcohol intake decreased. Regarding metabolic abnormalities, components of obesity and hyperglycemia worsened in men. In contrast, hyperglycemia was the only component found to have increased in women, whereas hypertension, hypertriglyceridemia, and low HDL cholesterol levels improved. Additionally, we investigated the characteristics of non-alcoholic participants (Additional file 1: Table S1). In non-alcoholic participants, both men and women showed the same trend of metabolic abnormalities as in the all participants.

**Table 1:** Characteristics of study participants at baseline examination

	Men			Women		
	Past (n = 4279)	Current (n = 8918)	*p *value	Past (n = 2502)	Current (n = 7361)	*p *value
Age (years old)	47.0 (16.0)	48.0 (16.0)	0.563	45.7 (10.1)	47.3 (14.0)	< 0.001
Body mass index (kg/m^2^)	23.0 (3.5)	23.5 (4.0)	< 0.001	21.7 (3.0)	21.4 (3.9)	< 0.001
Obesity	20.4 (870)	23.4 (2087)	< 0.001	11.7 (292)	10.5 (771)	0.096
Systolic blood pressure (mmHg)	122.8 (22.5)	121.3 (19.5)	< 0.001	115.6 (18.5)	112.3 (21.0)	< 0.001
Diastolic blood pressure (mmHg)	77.3 (14.5)	75.4 (15.5)	< 0.001	71.5 (11.2)	67.3 (15.0)	< 0.001
Hypertension	1.1 (48)	0.9 (82)	0.275	0.9 (23)	0.5 (34)	0.001
Fasting plasma glucose (mmol/L)	5.4 (0.7)	5.8 (0.7)	< 0.001	5.0 (0.9)	5.3 (0.6)	< 0.001
Hyperglycemia	21.6 (919)	42.2 (3759)	< 0.001	9.5 (237)	16.3 (1195)	< 0.001
Triglycerides (mmol/L)	1.6 (1.0)	1.1 (0.7)	< 0.001	1.0 (0.6)	0.7 (0.4)	< 0.001
Hypertriglyceridemia	33.5 (1432)	12.2 (1091)	< 0.001	9.4 (234)	2.3 (170)	< 0.001
High-density lipoprotein cholesterol (mmol/L)	1.2 (0.4)	1.5 (0.4)	< 0.001	1.4 (0.5)	1.9 (0.6)	< 0.001
Low high-density lipoprotein cholesterol levels	35.5 (1505)	7.2 (638)	< 0.001	37.4 (926)	6.0 (439)	< 0.001
Aspartate aminotransferase (IU/L)	21.8 (6.0)	19.1 (9.0)	< 0.001	18.6 (4.0)	15.5 (7.0)	< 0.001
Alanine aminotransferase (IU/L)	21.7 (12.0)	23.5 (13.0)	< 0.001	13.3 (6.0)	13.9 (14.8)	0.003
Gamma-glutamyltransferase (IU/L)	45.7 (34.0)	31.3 (33.4)	< 0.001	17.2 (10.0)	14.5 (6.5)	0.036
Smoking status						
Never smoker	21.6 (905)	37.2 (3269)	< 0.001	85.0 (2079)	85.9 (6285)	0.293
Ex-smoker	25.5 (1068)	33.4 (2941)	< 0.001	4.7 (116)	8.8 (646)	< 0.001
Current smoker	52.9 (2217)	29.4 (2585)	< 0.001	10.3 (251)	5.3 (389)	< 0.001
Habit of exercise	18.0 (771)	20.3 (1792)	0.002	18.4 (459)	16.2 (1176)	0.013
Alcohol consumption, g/week	131.4 (154.0)	95.4 (125.0)	< 0.001	27.3 (36.0)	24.9 (12.0)	0.172
Nonalcoholic	78.9 (3374)	85.2 (7602)	< 0.001	96.0 (2403)	95.2 (7009)	0.083
(None or minimal)	51.2 (2189)	63.4 (5656)	< 0.001	81.5 (2039)	84.7 (6234)	< 0.001
(Light)	27.7 (1185)	21.8 (1946)	< 0.001	14.5 (364)	10.5 (775)	< 0.001
Intermediate	15.3 (653)	10.8 (967)	< 0.001	2.8 (71)	2.9 (214)	0.858
Alcoholic	5.9 (252)	3.9 (349)	0.002	1.1 (28)	1.9 (138)	0.008
Fatty liver	22.5 (963)	31.2 (2785)	< 0.001	8.4 (211)	9.9 (721)	0.035
NAFLD	18.5 (792)	27.1 (2412)	< 0.001	8.0 (199)	9.4 (691)	0.024
NAFLD fibrosis score	− 2.27 (1.13)	− 2.38 (1.19)	< 0.001	− 2.22 (1.05)	− 2.60 (1.08)	< 0.001
Fib4 index	1.08 (0.51)	0.93 (0.53)	< 0.001	1.07 (0.45)	0.88 (0.47)	< 0.001

Data are expressed as median (IQR) or % (number) of subjects

*p* values by one-way analysis of variance for continuous variables and chi-squared test for categorical variables

BMI, body mass index; NAFLD, non-alcoholic fatty liver disease

Next, we compared the differences between patients with and without NAFLD in the past and current cohorts (Table 2). The prevalence of metabolic abnormalities and metabolic syndrome score was significantly higher in patients with NAFLD than in those without, for both genders, in both cohorts. Regarding regular exercisers, the ratio of regular exercisers was significantly lower among the patients with NAFLD than in those without, in both cohorts, for men. The ratio of regular exercisers was not different between the patients with NAFLD and those without in the two cohorts for women.

**Table 2:** Characteristics of study participants with and without NAFLD

	Past			Current			NAFLD+ Past versus current
	NAFLD– (n = 3487)	NAFLD+ (n = 792)	*p *value	NAFLD– (n = 6506)	NAFLD+ (n = 2412)	*p* value	*p *value
*Men*							
Age (years)	47.0 (16.0)	46.0 (15.0)	0.005	48.0 (17.0)	49.0 (14.0)	0.007	< 0.001
Body mass index (kg/m^2^)	22.4 (3.3)	25.0 (3.4)	< 0.001	22.3 (3.4)	25.3 (4.1)	< 0.001	0.140
Obesity	13.6 (474)	50.0 (396)	< 0.001	13.3 (863)	50.8 (1224)	< 0.001	0.716
Systolic blood pressure (mmHg)	119.5 (22.0)	125.5 (21.5)	< 0.001	119.0 (19.5)	124.5 (17.5)	< 0.001	< 0.001
Diastolic blood pressure (mmHg)	75.0 (14.5)	79.0 (15.0)	< 0.001	73.5 (15.5)	78.0 (14.0)	< 0.001	< 0.001
Hypertension	1.2 (41)	0.9 (7)	0.468	0.8 (54)	1.2 (28)	0.155	0.502
Fasting plasma glucose (mmol/L)	5.1 (0.7)	5.4 (0.9)	< 0.001	5.5 (0.6)	5.8 (0.8)	< 0.001	< 0.001
Hyperglycemia	18.7 (647)	34.5 (272)	< 0.001	36.6 (2382)	57.1 (1377)	< 0.001	< 0.001
Triglycerides (mmol/L)	1.3 (0.9)	1.9 (1.2)	< 0.001	0.8 (0.6)	1.2 (0.8)	< 0.001	< 0.001
Hypertriglyceridemia	28.2 (982)	56.8 (450)	< 0.001	8.0 (521)	23.6 (570)	< 0.001	< 0.001
High-density lipoprotein cholesterol (mmol/L)	1.2 (0.4)	1.0 (0.3)	< 0.001	1.6 (0.5)	1.3 (0.4)	< 0.001	< 0.001
Low high-density lipoprotein cholesterol levels	30.3 (1048)	57.9 (457)	< 0.001	4.4 (284)	14.7 (354)	< 0.001	< 0.001
Aspartate aminotransferase (IU/L)	20.0 (5.0)	23.0 (7.0)	< 0.001	16.0 (7.0)	19.0 (11.0)	< 0.001	< 0.001
Alanine aminotransferase (IU/L)	16.0 (10.0)	27.0 (20.8)	< 0.001	17.0 (9.0)	27.0 (21.0)	< 0.001	0.316
Gamma-glutamyltransferase (IU/L)	27.0 (32.0)	39.0 (39.0)	< 0.001	20.0 (17.0)	26.0 (19.0)	< 0.001	< 0.001
Smoking status							
Never smoker	21.0 (717)	24.4 (188)	0.042	36.0 (2310)	40.3 (959)	< 0.001	< 0.001
Ex-smoker	24.4 (835)	30.2 (233)	0.001	33.7 (2159)	32.9 (782)	0.491	0.163
Current smoker	54.6 (1866)	45.5 (351)	< 0.001	30.4 (1.947)	26.8 (638)	0.001	< 0.001
Habit of exercise	18.6 (649)	15.4 (122)	0.031	22.7 (1462)	13.9 (330)	< 0.001	0.287
Alcohol consumption, g/week	110.0 (208.0)	36.0 (110.0)	< 0.001	54.0 (179.0)	1.0 (54.0)	< 0.001	< 0.001
NAFLD Fibrosis Score	− 2.3 (1.1)	− 2.6 (1.1)	< 0.001	− 2.6 (1.1)	− 2.6 (1.1)	0.052	< 0.001
Fib4 index	1.1 (0.5)	0.9 (0.4)	< 0.001	0.9 (0.5)	0.8 (0.4)	< 0.001	0.035

	Past			Current			NAFLD+ Past versus current
	NAFLD– (n = 2303)	NAFLD+ (n = 199)	*p* value	NAFLD− (n = 6630)	NAFLD+ (n = 691)	*p* value	*p* value
*Women*							
Age (years)	45.0 (15.0)	52.0 (12.0)	< 0.001	46.0 (14.0)	53.0 (12.0)	< 0.001	0.696
Body mass index (kg/m^2^)	21.0 (3.5)	25.0 (4.4)	< 0.001	20.4 (3.4)	25.1 (5.0)	< 0.001	0.610
Obesity	8.5 (195)	48.7 (97)	< 0.001	6.6 (434)	48.3 (334)	< 0.001	0.919
Systolic blood pressure (mmHg)	110.5 (21.5)	126.5 (26.3)	< 0.001	109.0 (19.5)	124.0 (20.0)	< 0.001	< 0.001
Diastolic blood pressure (mmHg)	69.0 (13.0)	79.0 (15.8)	< 0.001	65.5 (14.0)	74.0 (15.0)	< 0.001	< 0.001
Hypertension	0.9 (20)	1.5 (20)	< 0.001	0.4 (27)	1.0 (7)	0.049	0.573
Fasting plasma glucose (mmol/L)	4.8 (0.7)	5.3 (0.9)	< 0.001	5.2 (0.5)	5.6 (0.7)	< 0.001	0.495
Hyperglycemia	7.7 (177)	30.3 (60)	< 0.001	13.2 (874)	44.7 (309)	< 0.001	< 0.001
Triglycerides (mmol/L)	0.9 (0.5)	1.4 (0.8)	< 0.001	0.5 (0.4)	1.0 (0.6)	< 0.001	< 0.001
Hypertriglyceridemia	7.6 (175)	29.8 (59)	< 0.001	1.3 (86)	12.0 (83)	< 0.001	< 0.001
High-density lipoprotein cholesterol (mmol/L)	1.4 (0.5)	1.2 (0.4)	< 0.001	1.9 (0.6)	1.5 (0.5)	< 0.001	< 0.001
Low high-density lipoprotein cholesterol levels	35.0 (799)	64.1 (127)	< 0.001	3.8 (251)	26.8 (185)	< 0.001	< 0.001
Aspartate aminotransferase (IU/L)	18.0 (4.0)	20.0 (5.0)	< 0.001	14.0 (6.0)	17.0 (9.0)	< 0.001	0.009
Alanine aminotransferase (IU/L)	11.0 (5.0)	19.0 (14.0)	< 0.001	12.0 (6.0)	19.0 (13.0)	< 0.001	0.367
Gamma-glutamyltransferase (IU/L)	11.0 (8.0)	19.0 (20.5)	< 0.001	13.0 (6.0)	17.0 (11.0)	< 0.001	0.006
Smoking status							
Never smoker	84.5 (1899)	90.9 (180)	0.010	85.9 (5663)	85.0 (585)	0.533	0.027
Ex-smoker	5.1 (115)	0.5 (1)	< 0.001	8.8 (580)	9.2 (53)	0.754	< 0.001
Current smoker	10.4 (234)	8.6 (17)	0.407	5.3 (349)	5.8 (40)	0.569	0.175
Habit of exercise	18.8 (432)	13.6 (27)	0.060	16.2 (1061)	14.8 (100)	0.345	0.664
Alcohol consumption, g/week	0.0 (36.0)	0.0 (0.0)	0.002	1.0 (12.0)	0.0 (1.0)	< 0.001	0.836
NAFLD fibrosis score	− 2.2 (1.0)	− 2.2 (1.1)	0.865	− 2.7 (1.0)	− 2.6 (1.1)	< 0.001	0.239
Fib4 index	1.1 (0.4)	1.0 (0.4)	0.009	0.8 (0.5)	0.8 (0.4)	0.070	0.008

Data are expressed as median (IQR) or % (number) of subjects

*p* values by one-way analysis of variance for continuous variables and chi-squared test for categorical variables

*BMI* body mass index, *NAFLD* non-alcoholic fatty liver disease

Then, we investigated the differences in the characteristics of NAFLD patients between the past and current cohorts. Regarding metabolic abnormalities, the prevalence of hyperglycemia increased, whereas the prevalence of hypertriglyceridemia and low HDL cholesterol levels decreased throughout these 2 decades for both genders. On the other hand, mean BMI was not different between the past and current cohort for both genders. Mean levels of AST and gamma-glutamyl transferase significantly decreased throughout the 2 decades for both genders. ALT levels did not change among genders. In addition, the proportion of exercisers decreased throughout these 2 decades for men and increased for women, although results were not statistically significant. Regarding alcohol consumption, that for men significantly decreased throughout these 2 decades, whereas that for women was not different. In addition, in men, the prevalence of fatty liver in none or minimal, light, and moderate and heavy alcohol consumer was 25.0% (547/2189), 20.7% (235/1185), and 18.9% (171/905) in the past cohort, and that in the current cohort was 33.9% (1917/5656), 25.4% (495/1946), and 28.3% (373/1316), respectively. In women, that in the past cohort was 8.6% (175/2039), 6.6% (24/364), and 12.1% (12/99), and that in the current cohort was 10.0% (624/6,234), 8.6% (67/775), and 8.5% (30/352), respectively. For men in the past cohort and women in the current cohort, as alcohol consumption increased, the prevalence of FLD tended to decrease, on the other hand, for men in the current cohort and women in the past cohort, light alcohol consumer tended to have a lower prevalence rate than the other groups. Additionally, the Fib4 index and NAFLD fibrosis score indicated that fibrosis was very mild.

The results of the multivariate analyses of factors related to NAFLD prevalence are presented in Table 3. Adjusting the OR for NAFLD prevalence for age was 0.99 (95% CI: 0.99–1.01, *p* = 0.894) in the past cohort, and 1.03 (1.03–1.04, p < 0.001) in the current cohort for men, whereas it was 0.99 (0.99–1.01, *p* = 0.979), and 1.04 (1.03–1.04, *p* < 0.001) in the past cohort, and 1.04 in the current cohort for women (1.03–1.04, *p* < 0.001). Alcohol consumption was negatively associated with the prevalence of NAFLD in both past and current cohorts in both genders. Exercise habit in the current cohort was negatively associated with the prevalence of NAFLD, and the tendency was shown in the past cohort as well, although results were not statistically significant. Adjusting the OR for NAFLD prevalence for BMI was 1.43 (1.37–1.50, *p* < 0.001) in the past cohort, and 1.37 (1.33–1.41, *p* < 0.001) in the current cohort for men, whereas for women, it was 1.33 (1.25–1.43, *p* < 0.001) in the past cohort and 1.39 (1.34–1.44, *p* < 0.001) in the current cohort. Similarly, adjusting the NAFLD prevalence the OR for obesity was 3.75 (3.01–4.66, *p* < 0.001) in the past cohort, and 4.36 (3.80–5.00, *p* < 0.001) in the current cohort for men. For women, the OR was 5.25 (3.59–7.67, *p* < 0.001) in the past cohort, and 8.35 (6.76–10.31, *p* < 0.001) in the current cohort.

**Table 3:** Multivariate analyses of factors associated with the prevalence of NAFLD

	Model 1				Model 2			
	Past		Current		Past		Current	
	OR (95%CI)	*p *value	OR (95%CI)	*p *value	OR (95%CI)	*p *value	OR (95%CI)	*p *value
*Men*								
Age, years old	0.99 (0.99–1.01)	0.894	1.03 (1.03–1.04)	< 0.001	0.99 (0.99–1.01)	0.979	1.04 (1.03–1.04)	< 0.001
Alcohol consumption, g/week	0.99 (0.98–0.99)	< 0.001	0.99 (0.98–0.99)	< 0.001	0.99 (0.99–0.99)	< 0.001	0.99 (0.98–0.99)	< 0.001
Regular exercise	0.86 (0.65–1.14)	0.289	0.74 (0.65–0.85)	< 0.001	0.85 (0.65–1.12)	0.258	0.66 (0.56–0.77)	< 0.001
Alanine aminotransferase (IU/L)	1.04 (1.04–1.05)	< 0.001	1.05 (1.05–1.06)	< 0.001	1.05 (1.04–1.06)	< 0.001	1.06 (1.06–1.07)	< 0.001
Ex-smoker	1.18 (0.89–1.56)	0.262	0.97 (0.83–1.13)	0.682	1.13 (0.86–1.49)	0.388	0.97 (0.84–1.12)	0.648
Current smoker	0.81 (0.62–1.06)	0.119	0.73 (0.62–0.86)	< 0.001	0.74 (0.57–0.95)	0.017	0.84 (0.72–0.98)	0.028
Body mass index, kg/m^2^	1.43 (1.37–1.50)	< 0.001	1.37 (1.33–1.41)	< 0.001	–	–	–	–
Systolic blood pressure, 1 mmHg	1.00 (0.99–1.01)	0.357	1.01 (1.00–1.01)	< 0.001	–	–	–	–
Fasting plasma glucose, 1 mmol/L	1.33 (1.19–1.48)	< 0.001	1.32 (1.20–1.46)	< 0.001	–	–	–	–
Triglycerides, 1 mmol/L	1.37 (1.24–1.52)	< 0.001	1.31 (1.19–1.45)	< 0.001	–	–	–	–
HDL-cholesterol, 1 mmol/L	0.49 (0.32–0.74)	< 0.001	0.36 (0.29–0.45)	< 0.001	–	–	–	–
Obesity	–	–	–	–	3.75 (3.01–4.66)	< 0.001	4.36 (3.80–5.00)	< 0.001
Hypertension	–	–	–	–	1.48 (0.56–3.93)	0.431	1.00 (0.53–1.87)	0.900
Hyperglycemia	–	–	–	–	1.71 (1.36–2.15)	< 0.001	1.76 (1.56–2.00)	< 0.001
Hypertriglyceridemia	–	–	–	–	2.49 (2.01–3.07)	< 0.001	2.61 (2.17–3.15)	< 0.001
Low high-density lipoprotein cholesterol levels	–	–	–	–	1.49 (1.21–1.84)	< 0.001	1.63 (1.30–2.04)	< 0.001
*Women*								
Age, years old	1.03 (1.00–1.05)	0.023	1.05 (1.03–1.06)	< 0.001	1.04 (1.02–1.06)	< 0.001	1.05 (1.04–1.06)	< 0.001
Alcohol consumption, g/week	0.99 (0.98–0.99)	< 0.001	0.99 (0.98–0.99)	< 0.001	0.99 (0.98–0.99)	< 0.001	0.99 (0.98–0.99)	< 0.001
Regular exerciser	0.73 (0.56–0.96)	0.217	0.89 (0.67–1.19)	0.427	0.69 (0.41–1.15)	0.153	0.75 (0.57–0.98)	0.035
Alanine aminotransferase (IU/L)	1.05 (1.05–1.06)	< 0.001	1.02 (1.01–1.03)	< 0.001	1.09 (1.07–1.12)	< 0.001	1.03 (1.02–1.04)	< 0.001
Ex-smoker	0.05 (0.01–0.52)	0.013	1.26 (0.87–1.82)	0.218	0.06 (0.01–0.51)	0.010	1.31 (0.93–1.84)	0.120
Current smoker	1.17 (0.63–2.19)	0.623	0.95 (0.59–1.52)	0.825	1.03 (0.55–1.92)	0.927	1.12 (0.73–1.73)	0.604
Body mass index, kg/m^2^	1.33 (1.25–1.43)	< 0.001	1.39 (1.34–1.44)	< 0.001	–	–	–	–
Systolic blood pressure, 1 mmHg	1.00 (0.99–1.01)	0.673	1.01 (1.01–1.02)	< 0.001	–	–	–	–
Fasting plasma glucose, 1 mmol/L	1.64 (1.27–2.11)	< 0.001	1.90 (1.55–2.33)	< 0.001	–	–	–	–
Triglycerides, 1 mmol/L	1.60 (1.19–2.14)	0.002	2.38 (1.89–3.00)	< 0.001	–	–	–	–
HDL-cholesterol, 1 mmol/L	0.39 (0.20–0.76)	0.005	0.33 (0.24–0.45)	< 0.001	–	–	–	–
Obesity	–	–	–	–	5.25 (3.59–7.67)	< 0.001	8.35 (6.76–10.31)	< 0.001
Hypertension	–	–	–	–	1.51 (0.27–8.33)	0.640	1.02 (0.33–3.19)	0.975
Hyperglycemia	–	–	–	–	2.33 (1.50–3.62)	< 0.001	2.91 (2.37–3.58)	< 0.001
Hypertriglyceridemia	–	–	–	–	1.70 (1.09–2.66)	0.020	2.69 (1.76–4.10)	< 0.001
Low high-density lipoprotein cholesterol levels	–	–	–	–	2.00 (1.37–2.91)	< 0.001	4.48 (3.38–5.94)	< 0.001

*CI* confidential interval, *HDL* high-density lipoprotein, *NAFLD* non-alcoholic fatty liver disease, *OR* odds ratio

**Fig. 2:**
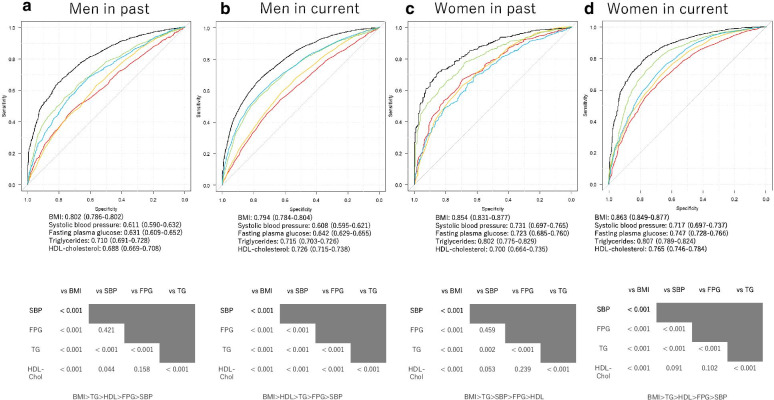
Area under the curve (AUC) for receiver operating characteristic (ROC) [95% confidence interval (CI)] of several factors for NAFLD incidence. Upper figure indicates ROC curve, AUC and cut-off value for each factor. Black line indicates body mass index (BMI); red line indicates systolic blood pressure (SBP); yellow line indicates fasting plasma glucose (FPG); green line indicates triglycerides (TG); and blue line indicates high density lipoprotein (HDL) cholesterol. Lower figure shows results of the comparison among each factor. **a** Men in the past cohort. **b** Men in the current cohort. **c** Women in the past cohort. **d** Women in the current cohort. **a** AUC for BMI was 0.802 (95% CI 0.786–0.802, *p* < 0.001), and that of SBP, FPG, TG, and HDL cholesterol was 0.611 (0.590–0.632, *p* < 0.001), 0.631 (0.609–0.652, *p* < 0.001), 0.710 (0.691–0.728, *p* < 0.001), and 0.688 (0.669–0.728, *p* < 0.001) in men of the past cohort, respectively. Cut-off value for BMI, SBP, FPG, TG, and HDL cholesterol was 23.2 kg/m^2^, 116.5 mmHg, 5.3 mmol/l, 1.3 mmol/l, and 1.0 mmol/l, respectively. **b** AUC for BMI was 0.794 (95% CI 0.784–0.804, *p* < 0.001), and for SBP, FPG, TG, and HDL cholesterol was 0.608 (0.595–0.621, *p* < 0.001), 0.642 (0.629–0.655, *p* < 0.001), 0.715 (0.703–0.726, *p* < 0.001), and 0.726 (0.715–0.738, *p* < 0.001) for men in the current cohort, respectively. Cut-off value for BMI, SBP, FPG, TG, and HDL cholesterol was 23.3 kg/m^2^, 118.5 mmHg, 5.6 mmol/l, 0.8 mmol/l, and 1.5 mmol/l, respectively. **c** AUC for BMI was 0.854 (95% CI 0.831–0.877, *p* < 0.001), and for SBP, FPG, TG, and HDL cholesterol was 0.731 (0.697–0.765, *p* < 0.001), 0.723 (0.685–0.760, *p* < 0.001), 0.802 (0.775–0.829, *p* < 0.001), and 0.700 (0.664–0.735, *p* < 0.001) for women in the past cohort, respectively. Cut-off value for BMI, SBP, FPG, TG, or HDL cholesterol was 22.4 kg/m^2^, 116.0 mmHg, 5.0 mmol/l, 1.2 mmol/l, and 1.3 mmol/l, respectively. **d** AUC for BMI was 0.863 (95% CI 0.849–0.877, *p* < 0.001), and for SBP, FPG, TG, and HDL cholesterol was 0.717 (0.697–0.737, *p* < 0.001), 0.747 (0.728–0.766, *p* < 0.001 vs. BMI), 0.807 (0.789–0.824, *p* < 0.001), and 0.765 (0.746–0.784, *p* < 0.001) for women in the past cohort, respectively. Cut-off value for BMI, SBP, FPG, TG, or HDL cholesterol was 22.5 kg/m^2^, 113.0 mmHg, 5.3 mmol/l, 0.7 mmol/l, and 1.6 mmol/l, respectively

Additionally, the ROC analyses and AUC for several risk factors for NAFLD prevalence are presented in Fig. 2. Comprehensively, in both genders for both cohorts, BMI was most significantly associated with the prevalence of NAFLD.

Since obesity was strongly associated with NAFLD prevalence, we investigated the differences in all patients’ characteristics with or without obesity and those who having NAFLD with and without obesity (Additional file 1: Table S2 and S3). BMI in the current cohort was significantly lower than that in the past cohort in non-obese patients in both genders. Non-obese, as well as obese men with NAFLD in the current cohort had a higher prevalence of hyperglycemia, with a lower prevalence of hypertriglyceridemia and low HDL cholesterol levels when compared with the past cohort. Non-obese women with NAFLD showed a similar tendency.

## Discussion

In this study, we showed that the prevalence of NAFLD significantly increased in both men and women throughout the 2 decades. The prevalence of hyperglycemia in NAFLD patients significantly increased through these 2 decades in men. For women, only the prevalence of hyperglycemia in NAFLD patients significantly increased throughout the 2 decades. Per capita food consumption has increased significantly in the world, from an average of 2360 kcal/person/day in the mid-1960s to 2800 kcal/person/day today, according to a study by the Food and Agriculture Organization of the United Nations [27]. Additionally, Wehmeyer et al. [28] reported that excessive calorie intake is associated with NAFLD. This increased caloric intake might be a significant contributor to the global rise of prevalence of NAFLD. On the other hand, in contrast to the global trend, the Japanese census showed a gradual decline in energy intake since its peak in 1975, as well as a decline in protein intake in women [29]. In men, the increased incidence of NAFLD despite the increased exercise habits may be due mainly to dietary intake changes not assessed in this study. The results of the Japanese census in 1996 showed that the daily fat intake for men, 63.0 ± 27.9 g, was significantly lower than that in 2018, 65.9 ± 29.2 g. In 1996, the daily fat intake for women, 55.2 ± 24.0 g, was not different from that in 2018, 55.5 ± 23.1 g [29]. These data suggest that the increase in NAFLD could be related to increased fat intake in men [30–32], whereas the main cause of increase in NAFLD in women could be due to the decreased exercise habits and not nutrient intake [33]. It was suggested that the lower mean BMI might be due to lower muscle mass [34]. In addition, Wada reported that waist circumference increased in the 55–69 age group of men throughout 2 decades [35]. Moreover, there were an increasing number of cases such as sarcopenia with increased visceral fat even though they were non-obese evaluated by BMI [36], which might be one of the reasons why the number of men with NAFLD is increasing, even though the BMI of men with NAFLD is not significantly different between past and present cohorts.

Among the metabolic syndrome components, obesity was most associated with the prevalence of NAFLD in men and women for both cohorts. A previous study reported the association between obesity and elevated circulating levels of pro-inflammatory factors, including cytokines or hormones, such as interleukin-1 and tumor-necrosis-factor. Lipid dysregulation in the liver, pro-inflammatory cytokines, and oxidative stress interact to promote fat accumulation in the liver [37]. Specifically, visceral fat accumulation is associated with NAFLD incidence because the venous splanchnic blood flow directly leads to a high exposure of liver tissue to increased free fatty acids and triglycerides by lipolysis [38]. On the other hand, triglycerides were lower and HDL-cholesterol was higher in men for current cohort than for the past cohort. Decreased serum triglycerides might be due to decreased alcohol consumption in patients with NAFLD. Increased HDL cholesterol might be due to increased prevalence of exercise.

In addition, the number of non-obese patients with NAFLD increased throughout these 2 decades. They became leaner than in the past cohort. However, the number of patients with impaired glucose tolerance increased. These results indicate that non-obese NAFLD and impaired glucose tolerance are closely associated with each other. We have previously reported that non-obese patients with NAFLD had a higher risk of diabetes than obese patients without NAFLD [21]. In the future, it is necessary to clarify the onset mechanisms of non-obese NAFLD.

Furthermore, the proportion of current smoker with NAFLD in the current cohort significantly decreased in men and tended to decrease in women. In addition, current smoking was negatively associated with the prevalence of NAFLD. Taken together, the decreased proportion of current smoker might be associated with the increased prevalence of NAFLD in the current cohort. Although several studies reported that smoking is a risk factor of incident NAFLD [39]^40^, it has been reported that smoking is not associated with the prevalence of NAFLD [41]^42^. Possible explanation is that smoking is associated with decreased food intake [43] and may decrease the prevalence of NAFLD. In addition, there is a possibility that the effect of the other risk factors on NAFLD might be higher than that of smoking.

The strengths of our study include using the same standardized diagnosis for fatty liver [12], standardized questionnaire for lifestyle factors, and the relatively large population-based longitudinal research design. Our study also has some limitations. Limited diagnostic accuracy to detect mild degree hepatic steatosis is taken as one limitation of abdominal ultrasonography for the evaluation of fatty liver [44], and liver steatosis was not assessed using the ultrasound parameters or scores. However, we assessed fatty liver on the basis of standardized diagnostic criteria, and we could have evaluated a certain level of fatty liver. A liver biopsy was required for a more accurate diagnosis for NAFLD and non-alcoholic steatohepatitis (NASH). In addition, self-reported alcohol intake might be inaccurate, especially heavy alcohol consumer, although several studies revealed that these consumption measures can be used with considerable confidence [45]^4647^. Second, we did not have data for plasma insulin concentrations to evaluate insulin resistance, such as homeostasis model assessment for insulin resistance. If we could evaluate insulin resistance, we could more accurately assess the relationship between alcohol intake and insulin resistance. Third, we did not have data for waist circumference, which are markers of visceral fat [48]. Thus, we cannot evaluate the obesity and NAFLD rigorously. Fourth, we also had a limited ability to examine different levels of physical activity. If we can assess the frequency and intensity of exercise, a more accurate analysis would be possible. Fifth, we did not have dietary data available. Sixth, several NAFLD-associated SNPs, such as rs738409, a single variant in the patatin-like phospholipase domain-containing protein 3, rs12447924 and rs12597002, cholesteryl ester transfer protein, rs58542926 C, a single nucleotide polymorphism in transmembrane 6 superfamily member 2, rs368234815 TT, the interferon lambda 4, and deficiency of the phosphatidylethanolamine N-methyltransferase [49–51]. Further investigation of SNPs in future studies will clarify the pathogenesis of NAFLD in the Japanese population. Moreover, compared to the total Japanese population, the participants in the past cohort study had fewer people under 29 and over 60 years old, and the participants in the current cohort had fewer people under 29 and over 65 years old. As the characteristics of the participants in this cohort study, most of them are annual medical examinations as instructed by their workplaces, therefore, the working-age population tends to be larger. Finally, the generalizability of our study to non-Japanese populations is uncertain.

## Conclusions

In conclusion, we showed that throughout these 2 decades, the prevalence of NAFLD significantly increased, and that obesity was the most associated factor with the prevalence of NAFLD. Thus, body weight management is essential as a treatment alternative for NAFLD. However, non-obese individuals and NAFLD also increased in these 2 decades. Therefore, it would have been much more solid a study analyzing trajectories on disease prevalence based un multiple cohorts instead of two cohorts taken some 20 years apart, and further studies targeting these non-obese NAFLD individuals are needed.

## Supplementary Information

**Additional file 1**. Summary of all additional Tables S1–S3. **Table S1**. Characteristics of non-alcoholic participants. **Table S2**. Characteristics of study participants with and without obesity. **Table S3**. Characteristics of study participants having NAFLD with and without obesity.

## Data Availability

The datasets used and analysed during the current study are available from the corresponding author on reasonable request.
