# Supplementary material for: Clinical characteristics and longitudinal changes of patients with non-alcoholic fatty liver disease in 2 decades: the NAGALA study
Source: BMC Gastroenterol. 2021 May 17;21:223. doi: 10.1186/s12876-021-01809-2 (PMC8130346; doi:10.1186/s12876-021-01809-2)
Supplement: Supplementary file 1 — Additional file 1. Summary of all additional Tables S1–S3. Table S1. Characteristics of non-alcoholic participants. Table S2. Characteristics of study participants with and without obesity. Table S3. Characteristics of study participants having NAFLD with and without obesity. [file 12876_2021_1809_MOESM1_ESM.docx]

**Clinical characteristics and longitudinal changes of patients with non-alcoholic fatty liver disease in two decades: the NAGALA study**

Takuro Okamura, MD, PhD^1^; Yoshitaka Hashimoto, MD, PhD^1^; Masahide Hamaguchi, MD, PhD^1^; Akihiro Obora, MD, PhD^2^; Takao Kojima, MD, PhD^2^; Michiaki Fukui, MD, PhD^1^

^1^ Department of Endocrinology and Metabolism, Kyoto Prefectural University of Medicine, Graduate School of Medical Science, Kyoto, Japan

^2^ Department of Gastroenterology, Asahi University Hospital, Gifu, Japan

Table S1. Characteristics of non-alcoholic participants

|  | Men | | | Women | |  |
| --- | --- | --- | --- | --- | --- | --- |
|  | Past  (n=3,374) | Current (n=7,602) | *p* - value | Past  (n=2,403) | Current  (n=7,009) | *p* - value |
| Age (years) | 46.6 (10.1) | 48.0 (10.4) | < 0.001 | 45.8 (10.1) | 47.3 (10.1) | < 0.001 |
| Body mass index (kg/m²) | 23.0 (2.9) | 23.5 (3.4) | < 0.001 | 21.7 (3.0) | 21.4 (3.4) | < 0.001 |
| Obesity | 21.5 (725) | 27.7 (2,106) | < 0.001 | 12.4 (298) | 12.9 (904) | 0.437 |
| Systolic blood pressure (mmHg) | 121.6 (17.2) | 120.3 (14.1) | < 0.001 | 115.4 (18.5) | 112.1 (15.2) | < 0.001 |
| Diastolic blood pressure (mmHg) | 76.3 (10.9) | 14.6 (11.0) | < 0.001 | 71.4 (11.1) | 67.1 (10.7) | < 0.001 |
| Hypertension | 29.0 (978) | 33.3 (2,509) | < 0.001 | 20.1 (483) | 17.2 (1206) | 0.001 |
| Fasting plasma glucose (mmol/L) | 5.4 (1.1) | 5.7 (1.0) | < 0.001 | 5.0 (0.9) | 5.3 (0.7) | < 0.001 |
| Hyperglycemia | 23.1 (779) | 46.8 (3,558) | < 0.001 | 10.9 (262) | 3.9 (273) | < 0.001 |
| Triglycerides (mmol/L) | 1.6 (1.0) | 1.0 (0.8) | < 0.001 | 1.0 (0.6) | 0.7 (0.4) | < 0.001 |
| Hypertriglyceridemia | 36.6 (1,235) | 14.6 (1,110) | < 0.001 | 12.0 (288) | 3.4 (238) | < 0.001 |
| High-density lipoprotein cholesterol (mmol/L) | 1.1 (0.3) | 1.5 (0.4) | < 0.001 | 1.4 (0.4) | 1.9 (0.4) | < 0.001 |
| Low high-density lipoprotein cholesterol levels | 39.1 (1,319) | 8.5 (646) | < 0.001 | 38.4 (923) | 6.9 (484) | < 0.001 |
| Metabolic syndrome score | 1.46 (1.28) | 1.29 (1.19) | < 0.001 | 0.92 (1.09) | 0.59 (0.92) | < 0.001 |
| Aspartate aminotransferase (IU/L) | 21.2 (6.7) | 18.6 (10.7) | < 0.001 | 18.6 (4.7) | 15.5 (13.1) | < 0.001 |
| Alanine aminotransferase (IU/L) | 21.5 (15.3) | 23.5 (17.0) | < 0.001 | 13.3 (8.5) | 14.5 (14.8) | < 0.001 |
| Gamma-glutamyltransferase (IU/L) | 38.2 (40.4) | 27.2 (23.6) | < 0.001 | 16.5 (20.5) | 15.8 (12.4) | 0.041 |
| Smoking status | - | - | < 0.001 | - | - | < 0.001 |
| Never smoker | 26.0 (877) | 39.9 (3,033) | < 0.001 | 86.5 (2,079) | 87.7 (6,147) | 0.029 |
| Ex-smoker | 26.0 (878) | 34.9 (2,653) | < 0.001 | 4.3 (103) | 7.9 (554) | < 0.001 |
| Current smoker | 48.0 (1,619) | 25.2 (1,916) | < 0.001 | 9.3 (223) | 4.4 (308) | < 0.001 |
| Habit of exercise | 18.6 (628) | 21.2 (127) | < 0.001 | 18.4 (442) | 16.9 (1,185) | 0.057 |
| Alcohol consumption, g/week | 64.6 (61.5) | 39.6 (53.4) | < 0.001 | 15.3 (29.9) | 11.3 (26.4) | < 0.001 |
| NAFLD Fibrosis Score | -2.30 (1.13) | -2.43 (1.17) | < 0.001 | -2.22 (1.05) | -2.61 (1.1) | < 0.001 |
| Fib4 index | 1.05 (0.46) | 0.89 (0.50) | < 0.001 | 1.07 (0.45) | 0.88 (0.48) | < 0.001 |
| Fatty liver | 23.5 (792) | 31.7 (2,412) | < 0.001 | 4.1 (199) | 9.9 (691) | < 0.001 |

Data are expressed as median (IQR) or % (number) of subjects.
*p* values by one-way analysis of variance for continuous variables and chi-squared test for categorical variables.

Table S2. Characteristics of study participants with and without obesity

| Men | | | | | | |  |  |  |  |  |  |
| --- | --- | --- | --- | --- | --- | --- | --- | --- | --- | --- | --- | --- |
|  | Obesity- | | | Obesity+ | | |  |  |  |  |  |  |
|  | Past  (n=3,409) | Current  (n=6,831) | *p* - value | Past  (n=870) | Current  (n=2,087) | *p* - value |  |  |  |  |  |  |
| Age (years) | 47.1 (9.9) | 48.5 (10.6) | < 0.001 | 46.4 (9.8) | 48.8 (9.9) | < 0.001 |  |  |  |  |  |  |
| Body mass index (kg/m²) | 21.9 (1.9) | 22.0 (1.9) | 0.921 | 27.0 (2.2) | 27.7 (2.7) | < 0.001 |  |  |  |  |  |  |
| Systolic blood pressure (mmHg) | 120.6 (16.8) | 119.0 (13.8) | < 0.001 | 130.7 (17.4) | 127.3 (13.6) | < 0.001 |  |  |  |  |  |  |
| Diastolic blood pressure (mmHg) | 75.9 (10.8) | 73.5 (10.7) | < 0.001 | 82.2 (11.2) | 80.3 (10.6) | < 0.001 |  |  |  |  |  |  |
| Hypertension | 6.3 (215) | 12.1 (827) | < 0.001 | 11.5 (100) | 25.1 (524) | < 0.001 |  |  |  |  |  |  |
| Fasting plasma glucose (mmol/L) | 5.3 (1.1) | 5.6 (0.8) | < 0.001 | 5.6 (1.3) | 6.2 (1.4) | < 0.001 |  |  |  |  |  |  |
| Hyperglycemia | 21.8 (743) | 42.0 (2,869) | < 0.001 | 35.4 (308) | 65.0 (1,357) | < 0.001 |  |  |  |  |  |  |
| Triglycerides (mmol/L) | 1.5 (1.1) | 1.0 (0.7) | < 0.001 | 2.0 (1.2) | 1.4 (1.1) | < 0.001 |  |  |  |  |  |  |
| Hypertriglyceridemia | 30.0 (1,023) | 10.0 (683) | < 0.001 | 52.2 (454) | 23.3 (486) | < 0.001 |  |  |  |  |  |  |
| High-density lipoprotein cholesterol (mmol/L) | 1.2 (0.3) | 1.6 (0.4) | < 0.001 | 1.1 (0.3) | 1.3 (0.3) | < 0.001 |  |  |  |  |  |  |
| Low high-density lipoprotein cholesterol levels | 31.4 (1,070) | 5.4 (369) | < 0.001 | 52.5 (457) | 14.9 (311) | < 0.001 |  |  |  |  |  |  |
| Metabolic syndrome score | 1.1 (1.0) | 0.9 (0.9) | < 0.001 | 2.9 (1.1) | 2.6 (1.0) | < 0.001 |  |  |  |  |  |  |
| Aspartate aminotransferase (IU/L) | 21.1 (9.0) | 17.7 (9.6) | < 0.001 | 24.4 (8.5) | 22.8 (13.7) | < 0.001 |  |  |  |  |  |  |
| Alanine aminotransferase (IU/L) | 19.2 (12.9) | 20.0 (12.4) | 0.003 | 30.5 (20.3) | 32.6 (22.3) | 0.006 |  |  |  |  |  |  |
| Gamma-glutamyltransferase (IU/L) | 42.7 (54.1) | 28.8 (32.5) | < 0.001 | 56.9 (49.8) | 37.9 (35.0) | < 0.001 |  |  |  |  |  |  |
| Smoking status |  | | | | | |  |  | |  | |  |
| Never smoker | 21.5 (733) | 32.7 (2,234) | < 0.001 | 23.9 (208) | 34.3 (716) | < 0.001 |  |  |  |  |  |  |
| Ex-smoker | 25.6 (873) | 37.1 (2,534) | < 0.001 | 29.4 (256) | 38.0 (793) | < 0.001 |  |  |  |  |  |  |
| Current smoker | 52.9 (1,803) | 27.3 (1,865) | < 0.001 | 46.7 (406) | 27.7 (578) | < 0.001 |  |  |  |  |  |  |
| Habit of exercise | 18.8 (641) | 23.1 (1,578) | 0.015 | 15.6 (136) | 17.8 (371) | 0.015 |  |  |  |  |  |  |
| Alcohol consumption, g/week | 130.6 (152.4) | 97.1 (149.4) | < 0.001 | 134.3 (173.6) | 90.8 (151.8) | < 0.001 |  |  |  |  |  |  |
| NAFLD Fibrosis Score | -2.3 (1.1) | -2.5 (1.1) | < 0.001 | -2.1 (1.2) | -2.0 (1.2) | < 0.001 |  |  |  |  |  |  |
| Fib4 index | 1.1 (0.5) | 0.9 (0.5) | < 0.001 | 1.0 (0.5) | 0.9 (0.5) | < 0.001 |  |  |  |  |  |  |
| Fatty liver | 15.1 (515) | 23.0 (1,571) | < 0.001 | 56.5 (492) | 70.8 (1,476) | < 0.001 |  |  |  |  |  |  |
| Women | | | | | | |  |  |  |  |  |  |
|  | Obesity- | | | Obesity+ | | |  |  |  |  |  |  |
|  | Past  (n=2,210) | Current  (n=6,590) | *p* - value | Past  (n=292) | Current  (n=771) | *p* - value |  |  |  |  |  |  |
| Age (years) | 45.2 (10.1) | 47.0 (10.1) | < 0.001 | 49.5 (9.2) | 49.6 (9.2) | 0.867 |  |  |  |  |  |  |
| Body mass index (kg/m²) | 20.9 (2.1) | 20.4 (2.2) | < 0.001 | 27.2 (2.1) | 27.9 (3.0) | < 0.001 |  |  |  |  |  |  |
| Systolic blood pressure (mmHg) | 113.5 (17.2) | 110.7 (14.4) | < 0.001 | 129.9 (20.6) | 123.1 (16.2) | < 0.001 |  |  |  |  |  |  |
| Diastolic blood pressure (mmHg) | 70.3 (10.6) | 66.3 (10.4) | < 0.001 | 79.7 (11.6) | 73.6 (11.3) | < 0.001 |  |  |  |  |  |  |
| Hypertension | 5.4 (119) | 5.4 (356) | 0.984 | 12.4 (36) | 20.6 (159) | < 0.001 |  |  |  |  |  |  |
| Fasting plasma glucose (mmol/L) | 4.9 (0.8) | 5.2 (0.5) | < 0.001 | 5.5 (1.5) | 5.8 (1.1) | < 0.001 |  |  |  |  |  |  |
| Hyperglycemia | 9.0 (199) | 16.1 (1,061) | < 0.001 | 25.7 (75) | 45.0 (347) | < 0.001 |  |  |  |  |  |  |
| Triglycerides (mmol/L) | 1.0 (0.5) | 0.6 (0.4) | < 0.001 | 1.4 (0.7) | 1.0 (0.7) | < 0.001 |  |  |  |  |  |  |
| Hypertriglyceridemia | 8.1 (179) | 2.1 (138) | < 0.001 | 25.2 (74) | 8.6 (66) | < 0.001 |  |  |  |  |  |  |
| High-density lipoprotein cholesterol (mmol/L) | 1.5 (0.4) | 1.9 (0.4) | < 0.001 | 1.2 (0.3) | 1.6 (0.4) | < 0.001 |  |  |  |  |  |  |
| Low high-density lipoprotein cholesterol levels | 34.5 (762) | 4.7 (310) | < 0.001 | 61.2 (179) | 20.0 (154) | < 0.001 |  |  |  |  |  |  |
| Metabolic syndrome score | 0.7 (0.9) | 0.4 (0.7) | < 0.001 | 2.6 (1.1) | 2.1 (1.0) | < 0.001 |  |  |  |  |  |  |
| Aspartate aminotransferase (IU/L) | 18.4 (4.4) | 15.1 (13.1) | < 0.001 | 20.1 (7.1) | 17.9 (11.7) | 0.001 |  |  |  |  |  |  |
| Alanine aminotransferase (IU/L) | 12.6 (7.3) | 13.6 (14.0) | 0.001 | 18.6 (13.1) | 20.9 (16.4) | 0.019 |  |  |  |  |  |  |
| Gamma-glutamyltransferase (IU/L) | 16.2 (21.9) | 15.4 (11.9) | 0.043 | 24.3 (24.9) | 21.7 (26.7) | 0.115 |  |  |  |  |  |  |
| Smoking status |  | | | | | |  | |  | |  | |
| Never smoker | 84.9 (1,876) | 86.5 (5,700) | 0.061 | 86.8 (253) | 83.8 (646) | 0.185 |  |  |  |  |  |  |
| Ex-smoker | 4.8 (106) | 8.5 (560) | < 0.001 | 3.9 (11) | 10.5 (81) | < 0.001 |  |  |  |  |  |  |
| Current smoker | 10.3 (228) | 5.0 (330) | < 0.001 | 9.3 (27) | 5.8 (44) | 0.027 |  |  |  |  |  |  |
| Habit of exercise | 19.3 (427) | 17.3 (1,140) | 0.031 | 12.4 (36) | 14.4 (111) | 0.349 |  |  |  |  |  |  |
| Alcohol consumption, g/week | 27.7 (88.6) | 25.2 (72.8) | 0.194 | 24.7 (66.4) | 22.3 (83.0) | 0.642 |  |  |  |  |  |  |
| NAFLD Fibrosis Score | -2.2 (1.0) | -2.7 (1.1) | < 0.001 | -2.0 (1.2) | -2.2 (1.2) | 0.039 |  |  |  |  |  |  |
| Fib4 index | 1.1 (0.5) | 0.9 (0.5) | < 0.001 | 1.0 (0.4) | 0.8 (0.5) | < 0.001 |  |  |  |  |  |  |
| Fatty liver | 5.4 (119) | 7.2 (474) | 0.001 | 36.7 (107) | 51.0 (393) | < 0.001 |  |  |  |  |  |  |

NAFLD, non-alcoholic fatty liver disease

Data are expressed as median (IQR) or % (number) of subjects.
*p* values by one-way analysis of variance for continuous variables and chi-squared test for categorical variables.

Table S3. Characteristics of study participants having NAFLD with and without obesity

| Participants with obesity | | | | | | |
| --- | --- | --- | --- | --- | --- | --- |
|  | Men | | | Women | | |
|  | Past  (n=396) | Current  (n=1,224) | *p* - value | Past  (n=97) | Current  (n=334) | *p* - value |
| Age (years) | 45.5 (15.5) | 48.0 (14.0) | 0.017 | 50.0 (12.0) | 52.0 (12.0) | 0.766 |
| Body mass index (kg/m²) | 26.9 (2.5) | 27.3 (3.3) | < 0.001 | 27.5 (3.2) | 27.8 (4.2) | 0.026 |
| Systolic blood pressure (mmHg) | 129.0 (23.8) | 127.0 (17.0) | < 0.001 | 130.5 (28.0) | 126.5 (19.4) | 0.030 |
| Diastolic blood pressure (mmHg) | 81.0 (16.0) | 80.0 (13.5) | 0.043 | 80.3 (15.1) | 75.8 (14.0) | 0.002 |
| Hypertension | 1.8 (7) | 1.6 (20) | 0.863 | 2.1 (2) | 1.2 (4) | 0.542 |
| Fasting plasma glucose (mmol/L) | 5.4 (0.9) | 5.9 (1.0) | < 0.001 | 5.3 (0.9) | 5.7 (0.9) | 0.894 |
| Hyperglycemia | 37.7 (148) | 63.5 (777) | < 0.001 | 29.9 (29) | 50.3 (168) | < 0.001 |
| Triglycerides (mmol/L) | 1.9 (1.3) | 1.2 (0.8) | < 0.001 | 1.4 (0.8) | 1.1 (0.6) | < 0.001 |
| Hypertriglyceridemia | 59.1 (234) | 27.5 (337) | < 0.001 | 30.9 (30) | 12.3 (41) | < 0.001 |
| High-density lipoprotein cholesterol (mmol/L) | 1.0 (0.3) | 1.2 (0.4) | < 0.001 | 1.1 (0.3) | 1.4 (0.4) | < 0.001 |
| Low high-density lipoprotein cholesterol levels | 61.8 (243) | 18.8 (230) | < 0.001 | 73.2 (71) | 32.6 (109) | < 0.001 |
| Aspartate aminotransferase (IU/L) | 24.0 (9.0) | 21.0 (13.0) | 0.095 | 20.0 (6.0) | 17.0 (10.8) | 0.469 |
| Alanine aminotransferase (IU/L) | 32.0 (24.0) | 31.0 (24.0) | 0.128 | 20.0 (15.0) | 21.0 (16.0) | 0.176 |
| Gamma-glutamyltransferase (IU/L) | 45.0 (40.5) | 29.0 (20.0) | < 0.001 | 20.0 (21.5) | 18.0 (12.0) | 0.066 |
| Smoking status |  | | | | | |
| Never smoker | 24.8 (95) | 39.8 (479) | < 0.001 | 93.8 (91) | 83.7 (278) | 0.007 |
| Ex-smoker | 28.5 (109) | 32.0 (386) | 0.186 | 0.0 (0) | 9.6 (32) | < 0.001 |
| Current smoker | 46.7 (179) | 28.2 (340) | < 0.001 | 6.2 (6) | 6.6 (22) | 0.876 |
| Habit of exercise | 13.6 (54) | 13.5 (163) | 0.943 | 13.4 (13) | 13.3 (43) | 0.974 |
| Alcohol consumption, g/week | 36.0 (110.0) | 1.0 (52.9) | < 0.001 | 0.0 (0.0) | 0.0 (1.0) | 0.598 |
| Participants without obesity | | | | | | |
|  | Men | | | Women | | |
|  | Past  (n=396) | Current  (n=1,188) | *p* - value | Past  (n=102) | Current  (n=357) | *p* - value |
| Age (years) | 47.0 (14.0) | 50.0 (14.0) | 0.002 | 53.0 (11.0) | 55.0 (11.0) | 0.416 |
| Body mass index (kg/m²) | 23.5 (1.8) | 23.3 (1.9) | < 0.001 | 32.2 (1.9) | 22.8 (2.3) | 0.016 |
| Systolic blood pressure (mmHg) | 121.5 (19.4) | 121.0 (18.0) | 0.006 | 124.5 (25) | 120.0 (20.3) | < 0.001 |
| Diastolic blood pressure (mmHg) | 77.0 (13.5) | 75.0 (14.0) | < 0.001 | 78.0 (16.0) | 72.5 (15.5) | < 0.001 |
| Hypertension | 0.0 (0) | 0.7 (8) | 0.032 | 1.0 (1) | 0.8 (3) | 0.895 |
| Fasting plasma glucose (mmol/L) | 5.3 (0.8) | 5.7 (0.7) | < 0.001 | 5.2 (0.8) | 5.6 (0.7) | 0.300 |
| Hyperglycemia | 31.4 (124) | 50.5 (600) | < 0.001 | 30.7 (31) | 39.5 (141) | 0.103 |
| Triglycerides (mmol/L) | 1.8 (1.2) | 1.1 (0.8) | < 0.001 | 1.4 (0.8) | 0.9 (0.6) | < 0.001 |
| Hypertriglyceridemia | 54.6 (216) | 19.6 (233) | < 0.001 | 28.7 (29) | 11.8 (42) | < 0.001 |
| High-density lipoprotein cholesterol (mmol/L) | 1.0 (0.3) | 1.3 (0.4) | < 0.001 | 1.2 (0.4) | 1.6 (0.5) | < 0.001 |
| Low high-density lipoprotein cholesterol levels | 54.0 (214) | 10.4 (124) | < 0.001 | 55.5 (56) | 21.3 (76) | < 0.001 |
| Aspartate aminotransferase (IU/L) | 22.0 (6.0) | 17.0 (9.0) | < 0.001 | 21.0 (5.0) | 16.0 (8.0) | < 0.001 |
| Alanine aminotransferase (IU/L) | 25.0 (16.0) | 23.0 (15.0) | 0.594 | 19.0 (12.0) | 18.0 (11.0) | 0.642 |
| Gamma-glutamyltransferase (IU/L) | 34.0 (31.0) | 24.0 (16.0) | < 0.001 | 19.0 (17.0) | 17.0 (10.0) | 0.040 |
| Smoking status |  | | | | | |
| Never smoker | 23.9 (93) | 40.9 (480) | < 0.001 | 88.1 (89) | 86.2 (307) | 0.619 |
| Ex-smoker | 31.9 (124) | 33.7 (396) | 0.500 | 1.0 (1) | 8.7 (31) | 0.002 |
| Current smoker | 44.2 (172) | 25.4 (298) | < 0.001 | 10.9 (11) | 5.1 (18) | 0.046 |
| Habit of exercise | 17.2 (68) | 14.3 (167) | 0.164 | 13.7 (14) | 16.2 (57) | 0.541 |
| Alcohol consumption, g/week | 36.0 (108.0) | 1.0 (54.0) | < 0.001 | 0.0 (0.0) | 0.0 (1.0) | 0.020 |

NAFLD, non-alcoholic fatty liver disease

Data are expressed as median (IQR) or % (number) of subjects.
*p* values by one-way analysis of variance for continuous variables and chi-squared test for categorical variables.
